# *Pulmonaria obscura* and *Pulmonaria officinalis* Extracts as Mitigators of Peroxynitrite-Induced Oxidative Stress and Cyclooxygenase-2 Inhibitors–In Vitro and In Silico Studies

**DOI:** 10.3390/molecules26030631

**Published:** 2021-01-26

**Authors:** Justyna Krzyżanowska-Kowalczyk, Mariusz Kowalczyk, Michał B. Ponczek, Łukasz Pecio, Paweł Nowak, Joanna Kolodziejczyk-Czepas

**Affiliations:** 1 Department of Biochemistry and Crop Quality, Institute of Soil Science and Plant Cultivation, State Research Institute, Czartoryskich 8, 24-100 Puławy, Poland; jkrzyzanowska@iung.pulawy.pl (J.K.-K.); mkowalczyk@iung.pulawy.pl (M.K.); lpecio@iung.pulawy.pl (Ł.P.); 2 Department of General Biochemistry, Faculty of Biology and Environmental Protection, University of Lodz, Pomorska 141/143, 90-236 Lodz, Poland; michal.ponczek@biol.uni.lodz.pl (M.B.P.); pawel.nowak@biol.uni.lodz.pl (P.N.)

**Keywords:** antioxidant, anti-inflammatory, blood plasma, cyclooxygenase-2, peroxynitrite, *Pulmonaria*, menisdaurin, salvianolic acid H

## Abstract

The *Pulmonaria* species (lungwort) are edible plants and traditional remedies for different disorders of the respiratory system. Our work covers a comparative study on biological actions in human blood plasma and cyclooxygenase-2 (COX-2) -inhibitory properties of plant extracts (i.e., phenolic-rich fractions) originated from aerial parts of *P. obscura* Dumort. and *P. officinalis* L. Phytochemical profiling demonstrated the abundance of phenolic acids and their derivatives (over 80% of the isolated fractions). Danshensu conjugates with caffeic acid, i.e., rosmarinic, lithospermic, salvianolic, monardic, shimobashiric and yunnaneic acids were identified as predominant components. The examined extracts (1–100 µg/mL) partly prevented harmful effects of the peroxynitrite-induced oxidative stress in blood plasma (decreased oxidative damage to blood plasma components and improved its non-enzymatic antioxidant capacity). The cellular safety of the extracts was confirmed in experimental models of blood platelets and peripheral blood mononuclear cells. COX-2 inhibitor screening evidently suggested a stronger activity of *P. officinalis* (IC_50_ of 13.28 and 7.24 µg/mL, in reaction with synthetic chromogen and physiological substrate (arachidonic acid), respectively). In silico studies on interactions of main components of the *Pulmonaria* extracts with the COX-2 demonstrated the abilities of ten compounds to bind with the enzyme, including rosmarinic acid, menisdaurin, globoidnan A and salvianolic acid H.

## 1. Introduction

The genus *Pulmonaria*, belonging to the Boraginaceae family, includes semi-evergreen perennials. Both *Pulmonaria officinalis* L. and *P. obscura* Dumort. species are edible plants and valuable ingredients of herbal medicines in Europe and western Asia. In the past, flowers and leaves of *Pulmonaria* sp. were used as wild plant food [1,2], and currently, these species have come back to our cuisine as components of vegan and vegetarian foods. Their aerial parts are also commercially available as *Pulmonariae Herba* or ingredients of various dietary supplements, herbal mixtures and tea. In Poland, *Pulmonariae Herba* is registered in the National Database of Health Protection Products and approved for pharmaceutical use [3]. The common name of *Pulmonaria*, i.e., “lungwort”, derives from its traditional medicine recommendations such as treatment of disorders related to the pulmonary system (e.g., bronchitis, cold, cough, laryngitis and sore throat). Furthermore, data from numerous ethnomedicinal studies indicate that infusions and decoctions based on these plants may be also useful in the treatment of other complaints [4,5,6,7,8,9]. In contemporary medicine, *Pulmonariae Herba* is primarily used to alleviate pulmonary disorders, as an expectorant, anti-inflammatory and mucilaginous drug. Externally, lungwort is applied to heal burns, wounds, cuts and eczema [10,11,12].

The present work is the first study on biological properties of *P. obscura* and *P. officinalis*, covering their activities in human blood plasma under the peroxynitrite-induced oxidative stress, verification of cellular safety and preliminary evaluation of anti-inflammatory potential (based on the COX-2 inhibitory tests). Despite the presence of *Pulmonaria* species in ethnomedicine and their use for culinary purposes, the biological activity of the lungwort-based preparations and extracts from these plants is still poorly recognized. Moreover, there is a lack of comparative studies assessing the bioactive properties of different species from the *Pulmonaria* genus. Most of the available evidence derived from strictly chemical assays, mostly based only on DPPH^•^(1,1-diphenyl-2-picrylhydrazyl) or ABTS^•+^ [2,2′-azino-*bis*(3-ethylbenzothiazoline-6-sulphonic acid)] radical scavenging assays [13]. Furthermore, very few studies on these plants were conducted with the use of biological experimental models such as biomolecules, blood cells, or any cell lines. For example, recent studies of Neagu et al. [14] have provided data on acetylcholinesterase and tyrosinase inhibitory actions of aqueous and ethanolic extracts obtained from *P. officinalis*. It has been also shown that the *P. officinalis*-derived fraction may significantly decrease the synthesis of α-toxin in *Staphylococcus aureus*, slightly weaken the expression of staphylococcal protein A and inhibit the activity of staphylococcal sortase A [15].

The activity of *P. obscura* and *P. officialis* extracts was examined in a panel of different bioassays, including in vitro and in silico analyses. To the best of our knowledge, the influence of *Pulmonaria* extracts on the physiology of the cardiovascular system, including antioxidant action towards peroxynitrite (ONOO^−^), one of the primary physiological oxidants, and their effects on components of blood plasma have not been described yet. Since the in-vivo formation of ONOO^−^ is strictly related to the pathophysiology of inflammation, inhibitory effects of the extracts on a pro-inflammatory enzyme, cyclooxygenase-2 (COX-2) were also evaluated. Thus, our study was based on the following questions: *Can the examined extract reduce oxidative damage to blood components*? *Do they have any anti-inflammatory potential*? Additionally, the risk of cytotoxicity of the examined plant preparations towards blood cells (incl. blood platelets and peripheral blood mononuclear cells, PBMCs) was evaluated.

## 2. Results

### 2.1. Phytochemical Profiling

**Figure 1:**
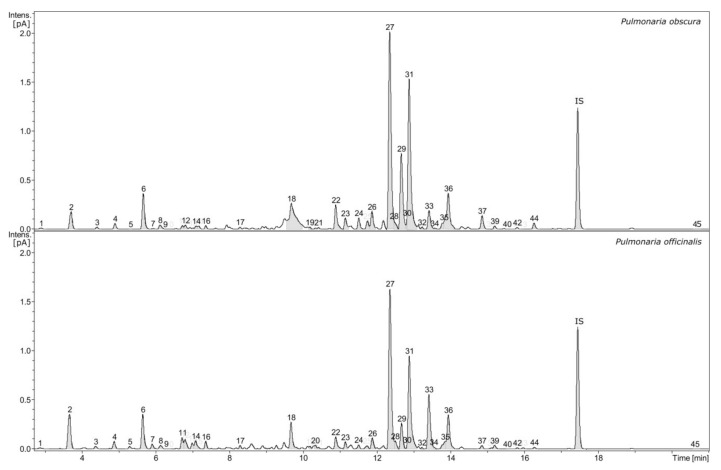
Chromatographic profile of *Pulmonaria obscura* and *Pulmonaria officinalis* phenolic-rich fractions (signal form charged aerosol detector, numbers indicate compounds that were characterized and quantitatively analyzed, IS–internal standard).

High-resolution mass spectrometry (HR-MS) analysis revealed the presence of numerous phenolic compounds; some of these compounds have well-documented biological properties. The phytochemical profiles of the examined extracts - i.e., phenolic-rich fractions obtained from *P. officinalis* and *P. obscura* were very similar, both qualitatively and quantitatively (Figure 1).

The examined fractions (extracts) contained several compounds belonging to various classes of metabolites. However, in both extracts, the most-represented substances were members of the broadly defined group of phenolic acids, constituting over 81% of fractions. The estimated content of individual groups of specific metabolites was presented in Table 1. The dominant compounds were conjugates of danshensu with caffeic acid, i.e., rosmarinic, lithospermic, salvianolic, monardic, shimobashiric and yunnaneic acids. The presence of esters of caffeic acid with quinic acid (so-called chlorogenic acids), threonic and glyceric acids was also revealed. Three isomers of *p*-coumaroylquinic acid, a few lignans such as globoidnans A and B, pulmonariosides A and B as well as many common flavonol glycosides such as quercetin and kaempferol derivatives (including malonylated forms) were also found. Furthermore, the examined fractions contained a significant content of menisdaurin, a nitrile glucoside as well as slight amounts of megastigmane glucoside actinidioionoside and tryptophan derivative, i.e., lycoperodine-1 [16].

The results of our quantitative analyses of *P. obscura* and *P. officinalis* phenolic-rich fractions are shown in Table 2. Menisdaurin (**2**), caffeic acid (**8**), salvianolic acid H (**33**), glyceric acid (**11**, **14**) and threonic acid (**4, 12**) derivatives were present in higher concentrations in *P. officinalis* extract. In contrast, in the *P. obscura* extract, the increased concentrations of yunnaneic acid E (**22**), rosmarinic acid (**27**), monardic acid (**29**), lithospermic acid A (**31**) and globoidnan A (**37**) were observed.

### 2.2. Antioxidant Assays

#### 2.2.1. ONOO^−^-Scavenging Ability in the Evans Blue Solution

The preliminary step of our studies involved a comparative evaluation of the ONOO^−^-scavenging abilities of *Pulmonaria* phenolic-rich fractions in a non-biological experimental system (i.e., a solution of Evans blue dye). The assay revealed similar abilities of both of these plant-derived preparations to scavenge 1 mM ONOO^−^. Their efficiencies were characterized by IC_50_ values of 36.71 µg/mL and 32.66 µg/mL, for *P. obscura* and *P. officinalis*, respectively. Furthermore, the fractions were noticeably weaker ONOO^−^ scavengers, when compared to reference antioxidants such as rosmarinic acid and Trolox^®^ (Table 3).

#### 2.2.2. Antioxidant Activity in Human Blood Plasma

**Figure 2:**
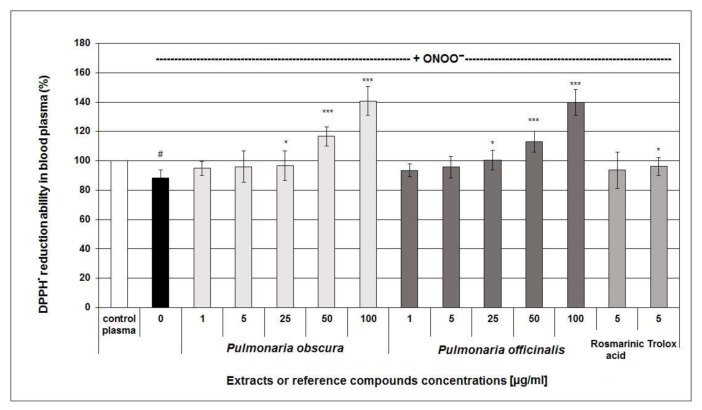
Protective effect of the examined *Pulmonaria* extracts (50% methanolic fractions) on the non-enzymatic antioxidant capacity of blood plasma under the 150 µM ONOO^−^-induced oxidative stress. The assay was based on DPPH^•^ radical scavenging ability as a biomarker of the non-enzymatic antioxidant capacity of blood plasma. DPPH-scavenging efficiency of control (untreated) plasma was assumed as 100% of NEAC; (* *p* < 0.05; *** *p* < 0.001 for plasma treated with ONOO^−^ in the absence of examined substances versus plasma treated with ONOO^−^ in the presence of the extracts; ^#^ *p* < 0.05 for the control plasma vs. samples treated with ONOO^−^ in the absence of examined substances; *n* = 6).

In the next part of the study, in-vitro experiments employing human blood plasma exposed to 100 or 150 μM ONOO^−^ providing more promising results and demonstrated that the examined *Pulmonaria* phenolic-rich-fractions might display antioxidant effects in biological experimental systems. The investigated *Pulmonaria* fractions partly prevented the ONOO^−^-induced decrease of the non-enzymatic antioxidant capacity of human blood plasma (NEAC; Figure 2). A slight effect on NEAC was observed for all the tested concentrations of *Pulmonaria* extracts; however, its statistical significance was found for their concentrations of 25–100 µg/mL. Furthermore, at concentrations of 50–100 µg/mL, the phenolic-rich fractions significantly enhanced the NEAC of blood plasma, compared to control samples (Figure 2). However, no statistical significance between the efficiency of *P. obscura* and *P. officinalis* extracts was found (*p* > 0.05).

Antioxidant efficacies of the examined plant preparations were also confirmed by determination of the levels of protein and lipid biomarkers of oxidative stress, i.e., 3-nitrotyrosine (3-NT), lipid hydroperoxides and the thiobarbituric acid-reactive substances (TBARS) in blood plasma. The obtained results indicated that both of the examined *Pulmonaria* extracts were more potent inhibitors of plasma lipid peroxidation than protein nitration. Some anti-nitrative tendency was observed for most of the tested concentrations (at 1–100 µg/mL); however, a statistically significant reduction of 3-nitrotyrosine level blood plasma proteins and isolated fibrinogen was mostly found only for higher concentrations of the extracts (i.e., 25–100 µg/mL) (Table 4).

The ONOO^−^-induced peroxidation of plasma lipids was significantly diminished in the presence of *Pulmonaria* fractions, even at their lowest concentrations (i.e., at concentrations ≤ 5 µg/mL) (Table 5).

### 2.3. COX-2 Inhibitor Screening Tests

In order to preliminary assess whether the examined *Pulmonaria* phenolic-rich fractions display any anti-inflammatory potential, a key inflammatory enzyme, i.e., COX-2 was used. In ELISA, the COX-2-dependent metabolism of arachidonic acid was evidently reduced by the *P. officinalis* fraction; the *P. obscura* extract had a weaker inhibitory action on this enzyme (Table 6). While the IC_50_ for *P. officinalis* extract was 7.24 μg/mL, the IC_50_ for *P. obscura* fraction attained 51 μg/mL in this test. The colorimetric assay confirmed a higher COX-2-inhibitory potency of the fraction originated from *P. officinalis*, when compared to the *P. obscura* extract. For indomethacin (a reference COX inhibitor and anti-inflammatory drug), the IC_50_ values were estimated to be 0.05 and 2.06 μg/mL, for the ELISA and colorimetric assay, respectively (Table 6).

### 2.4. Assessments of Cellular Safety

The possibility of toxic action of the examined extracts towards blood cells was evaluated using three different experimental systems. The risk of direct damage to cell membranes was excluded using propidium iodide assay. No cytotoxicity towards PBMCs was found in this assay for both of the examined *Pulmonaria* preparations. Furthermore, the resazurin-based viability test excluded the inhibitory influence of *Pulmonaria* fractions on the metabolic activity of PBMCs. Cellular safety of the extracts was also confirmed in experiments on blood platelets. Due to susceptibility to damaging factors and very high reactivity in a response to different stimuli, blood platelets are very good indicators in various cytotoxicity tests. Thus, the platelet LDH-leakage assays provided additional evidence of the cellular safety of *Pulmonaria* extracts in vitro (Table 7).

### 2.5. Prediction of Protease Inhibitors, Molecular Docking and Drug-Likeness

Based on their dominant contents (≥4 µg/mg of the fraction), fourteen main components of the examined *Pulmonaria* extracts were selected for in silico analyses. The use of predictive bioinformatics tools allowed us to analyze the possibility of interaction of the individual compounds with the COX-2 enzyme, based on mathematical analyses and energy calculations. Predict Bioactivity Cheminformatics Tool and the Vina dockings showed that ten compounds could be probable inhibitors of COX-2 in *Pulmonaria* plants (i.e., 2-*O*-*E*-caffeoyl-l-threonic acid (**4**), chlorogenic acid (**6**), globoidnan A (**37**), globoidnan B (**18**), lithospermic acid A (**31**), menisdaurin (**2**), monardic acid (**29**) rosmarinic acid (**27**), quercetin 3-*O*-(6’’-*O*-malonyl)-β-glucoside (**23**) and salvianolic acid H (**33**)) (Table 8).

**Figure 3:**
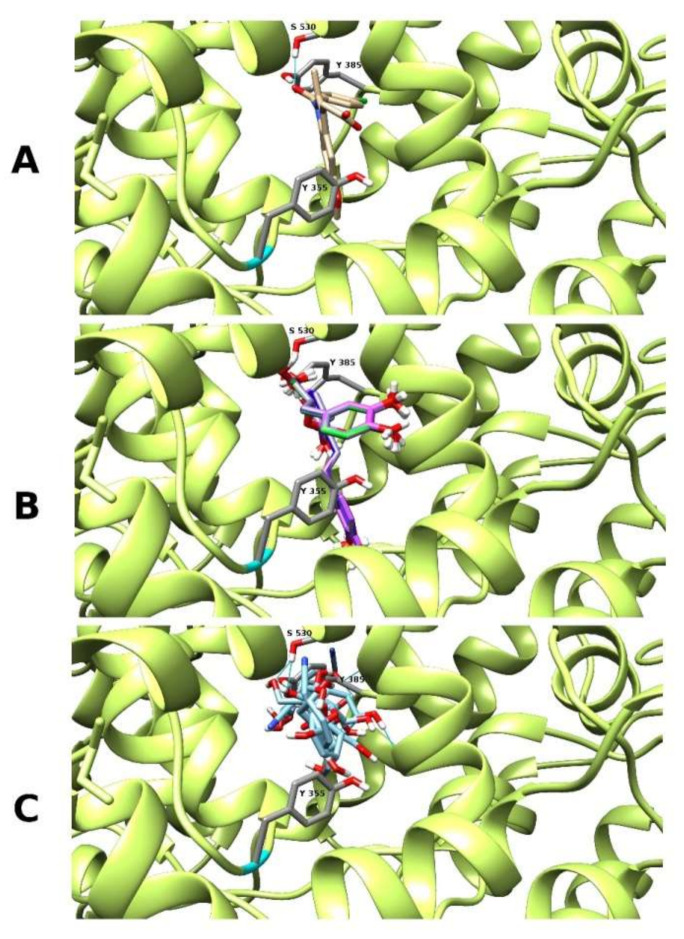
The molecular docking of menisdaurin (**C**) and salvianolic acid H (**B**) with comparison to reference compound indomethacin (**A**), according to its crystal localization.

However, only two of them were definitely more abundant in the *P. officinalis* fractions (Table 2), where the inhibition of COX–2 activity was much stronger compared to the fraction obtained from *P. obscura* (Table 6). These two compounds were menisdaurin (**2**) and salvianolic acid H (**33**), and their free energy change of binding (ΔG**°_bind_**) was much lower than values counted for indomethacin indicating on their stronger affinity when compared to this reference inhibitor. Both compounds were bound in a similar location as indomethacin in COX–2 active site between amino acid residues Tyr 355, Tyr 385 and Ser 530, according to human protein structure numbering (Figure 3).

## 3. Discussion

Natural, plant–derived substances with antioxidant activity are helpful and promising agents in the prevention of different civilization disorders. On the other hand, the newest findings have indicated that the antioxidant–based action solely may be insufficient in the prophylaxis of these diseases [17,18,19]. Due to multifactorial pathomechanisms of Western diseases, there is a need to find substances with a broader spectrum of beneficial activities, including both antioxidant and anti-inflammatory effects. The contribution of inflammation and oxidative stress to the pathogenesis and progression of a wide range of diseases, including cardiovascular disorders, has been well documented and described in numerous papers [20,21,22,23]. The co–existence of inflammation and oxidative stress in the cardiovascular system results in damage to the vessel wall, blood cells and non-cellular blood components, leading to undesirable changes in their biological activities. The NADPH oxidase subunits p47phox, Nox1, Nox2 and Nox4, mitochondrial ROS as well as enzymatic activities of xanthine oxidase and uncoupled eNOS are the primary sources of oxidants generated within the cardiovascular system [24].

Among a variety of the reactive oxygen species that are generated within the cardiovascular system, the formation of ONOO^−^ is particularly important in the pathophysiology of stroke, myocardial infarction, chronic heart failure, diabetes, circulatory shock, chronic inflammatory diseases, cancer as well as neurodegenerative disorders development [25]. Peroxynitrite can modify both proteins and lipids. It has been established that this potent oxidative and nitrative agent may augment [26,27] or diminish [28] coagulant activity of fibrinogen; its pro-thrombotic effects may be also a result of modifications of fibrinolytic proteins [29,30].

The present work is the first study devoted to a comparative evaluation of antioxidant activity, the COX–2 inhibitory potential and cellular safety of extracts obtained from *Pulmonaria obscura* and *P. officinalis*. The preliminary part of this work, i.e., the ONOO^−^ scavenging assay was based on the Evans blue decolorization and suggested moderate antioxidant effects of the *Pulmonaria* phenolic–rich fractions (IC_50_ = 36.71 and 32.66 µg/mL), compared to well–known antioxidants, i.e., rosmarinic acid (IC_50_ = 13.34 µg/mL) or Trolox (IC_50_ 2.76 µg/mL). According to the literature, the antioxidant activity of plant extracts or individual plant–derived compounds in this test may differ drastically. In our earlier studies, the yunnaneic acid B isolated from *P. officinalis* scavenged ONOO^−^ with the IC_50_ = 50.45 μg/mL [31]. The efficiency of both *Pulmonaria* extracts was significantly higher compared to some other plant extract, e.g., the *Newbouldia laevis* leaf extract, which was able to scavenge ONOO^−^ with IC_50_ = 1210.83 µg/mL [32]. On the other hand, in studies on *Diplazium esculentum* extract, the IC_50_ value for this plant was 3.35 µg/mL, whereas the IC_50_ for gallic acid amounted to 0.87 µg/mL [33]. However, this common screening assay required the use of a 1 mM concentration of ONOO^−^, which is unachievable in vivo. Therefore, in further steps of the study (experiments on isolated fibrinogen and blood plasma), ONOO^−^ was used at significantly lower concentrations (of 100 or 150 µM). Due to instability of peroxynitrite at physiological pH, the use of 100–150 µM concentration reflects physiologically relevant levels of this oxidant, that may be formed in vivo, e.g., at sites of inflammation. According to literature, a bolus addition of 250 µM ONOO^−^ corresponds to a steady–state level of its 1 µM concentrations, maintained for 7 min [34].

Biological activities of the examined plant extracts were analyzed in vitro, employing a model experimental system of blood plasma or isolated protein. Our results obtained from experiments in blood plasma mostly revealed comparable antioxidant efficiencies of both of the examined *Pulmonaria* preparations. The extracts slightly reduced the impairment of the NEAC of blood plasma caused by ONOO^−^ and decreased the ONOO^−^–induced formation of 3–nitrotyrosine in blood plasma proteins. The lipoperoxidation assays provided demonstrated significant protective effects of the examined *Pulmonaria* phenolic–rich fractions at most of the tested concentrations. However, the above lipid peroxidation assays have some important limitations in the context of their use in this type of study. Both of them are only partly specific to lipoperoxidation products. In our studies, a reduction of plasma lipid peroxidation was detectable only into a level higher than 50%, in a full range of the tested concentrations of the *Pulmonaria* extracts (1–100 µg/mL), with no dose–dependent activity of the extracts in most cases. Additionally, at the concentrations of the extracts of 50–100 µg/mL, a plateau phase of the reaction was observed, probably as a result of non-specific interactions (interference) of the extract components with reagents and other components of the assayed blood plasma samples.

Our phytochemical analyses and antioxidant assays suggested that the *Pulmonaria* extracts may be sources of natural antioxidants. The examined extracts contained over 80% phenolic acids and about 5% flavonols. In a context of biological activity (including antioxidant properties) of *Pulmonaria* extracts, the presence of considerable amounts of rosmarinic acid and its derivatives may be particularly important. With a content of 301.46 and 159.28 µg/mg, this compound was the predominant component of *P. obscura* and *P. officinalis* fractions, respectively. Its ability to scavenge reactive oxygen species or to prevent their generation has been confirmed by different research groups [35]. Rosmarinic acid has been described as an effective reducer of peroxynitrite–mediated damage, both at the level of peroxynitrite scavenging and inhibition of superoxide and nitric oxide synthesis [36]. Furthermore, our earlier studies [31], demonstrated significant protective activity of its derivative, i.e., yunnaneic acid B (also isolated from *P. officinalis*) against oxidative and nitrative damage induced by peroxynitrite. It is very likely that the presence of the oligomeric derivatives of rosmarinic acid such as yunnaneic acids B and E, in the examined *Pulmonaria* extracts also might contribute to their antioxidant effects.

Although in ethnomedicine *Pulmonaria* plants are used to alleviate inflammation, neither detailed information on their anti-inflammatory efficiency nor comparative assessments of biological activities of different species exist. As a key pro-inflammatory enzyme and target of many non-steroidal anti-inflammatory drugs, the COX–2 enzyme was used in our tests. Contrary to results indicating similar antioxidant activities of both extracts, our COX–2 inhibitor analyses revealed noticeable divergences in their effects. In the case of both the detection of the arachidonic acid metabolite (PGF2α) and reaction with a synthetic substrate, inhibitory actions of the extracts on COX–2 activity clearly indicated a stronger effect of the *P. officinalis* extract (IC_50_ of 7.24 and 13.28 µg/mL, respectively). The COX–2 inhibitory action of *P. obscura* extract was lower; its IC_50_ amounted to 51.00 and 58.59 µg/mL in the ELISA and colorimetric test, respectively. According to the available literature, plant extracts may inhibit this enzyme with a wide range of efficiency. For example, in studies of Ondua et al. [37], the IC_50_ for *Plantago lanceolata* leaf extracts was 0.41 μg/mL. In other work, a leaf extract prepared from *Eucomis autumnalis* ssp. *autumnalis* reduced the COX–2 activity with the IC_50_ of 29.00 μg/mL [38]. Moreover, the activity of fractions originated from the same plant material also may be divergent, e.g., for two ethanolic fractions isolated from *Stachys officinalis* extract undergoing the micro- or nanofiltration, the IC_50_ values amounted to 38.8 and 1.2 μg/mL, respectively. The IC_50_ for quercetin (a reference compound) was 35.2 μg/mL [39].

Our invitro screening of the COX–2 inhibitory activity was additionally supported by in silico studies on possible interactions of main components of the extracts and the enzyme. Biochemical mechanisms of anti-inflammatory action of these plants are still inadequately recognized. However, based on current knowledge of the anti-inflammatory effects of hydroxycinnamates such as rosmarinic and chlorogenic acids, it may be predicted that their presence considerably contributed to the COX–2 inhibitory properties of the examined plant extracts. The anti-inflammatory action of rosmarinic acid, involving different pathways of pro-inflammatory response (such as inhibition of NFκB activation, suppression of the complement cascade and reduction of pro-inflammatory cytokines as well as the inhibitory effect on COX–2) was observed in vitro and in vivo [40]. Similarly, chlorogenic acid also displays an anti-inflammatory effect [41]. Besides the aforementioned phenolic acids, the anti-inflammatory action or COX–inhibitory properties of other main constituents of the examined extracts has been described to a significantly lower extent. However, also other phytochemicals may be responsible for stronger inhibition of the COX–2 by the *P. officinalis* extract. Although no information on the anti-inflammatory properties of globoidnan A and B is available, monardic acid A was found to inhibit hyaluronidase activity and moderately reduce histamine release in vitro [42]. The anti-inflammatory activity of shimobashiric acid B has been recently described [43], but there is no such evidence for its C isomer, present in the examined fractions from *Pulmonaria* species. The COX–2 inhibitory activity of menisdaurin was demonstrated in a study by Muhammad and Sirat [44]. In analyses of COX–2 inhibitors, isolated from the stem bark of *Bauhinia rufescens*, menisdaurin inhibited the enzyme with the IC_50_ value of 72.28 μM (22.6 μg/mL), whereas the IC_50_ for the positive control, indomethacin was of 0.24 μM (0.085 μg/mL). Our in vitro and in silico observations are in agreement with the cited results and indicate that menisdaurin (39.08 ± 1.56 µg/mg of the fraction, Table 2), which is more abundant in *P. officinalis*, may be a potent inhibitor of COX–2. However, it should be emphasized that this compound was detected only in *Pulmonaria* samples collected during the spring, whereas samples collected in the autumn did not contain menisdaurin at all [16]. The other significant inhibitor of COX–2 in *P. officinalis* phenolic–rich fraction may be salvianolic acid H (26.23 ± 0.75 µg/mg of the extract) as we also showed its strong affinity to COX–2 active site in molecular docking (Table 8, Figure 3A). According to the UniProt P35354 record, the Tyr 324, Tyr 353 and Ser 499 residues are important for COX–2 enzymatic function (Figure 3). Tyr 321 is substrate binding site, Tyr 353 is the cyclooxygenase active site, and Ser 499 is the aspirin–acetylated site. Indomethacin and other non-steroidal anti-inflammatory drugs bind there as COX–2 inhibitors (Figure 3C). Salvianolic acid H and menisdaurin are bound there according to our molecular docking studies, which can confirm that they are important COX–2 inhibitor compounds in *P. officinalis*, responsible to a large extent for the observed effect of a stronger inhibition of COX–2 enzyme activity by extract isolated from this plant species.

The presented work provides some evidence of the bioactivity of extracts from *P. obscura* and *P. officinalis*. The obtained results suggested that both plants are a source of phytochemicals with antioxidant and cyclooxygenase-inhibitory activity in vitro; however, the observed activity may not be maintained in vivo. The evaluation of the physiological relevance of these effects requires further, more advanced tests, including in vivo studies and experiments on the digested/hydrolyzed plant material. Biological activity (including the mechanisms of antioxidant action) of the digested extracts may differ from the reactive oxygen species scavenging properties of the non-hydrolyzed substances. Contemporary use of plant–derived substances includes their external and internal applications. Herbal ointments, balms and gels are used in the treatment of dermatological disorders, e.g., to alleviate inflammation and to heal skin irritations and wounds of different etiology. In the case of external applications of plant material, indigested extracts might be active, but in the case of internal use, many additional aspects of their activity should be established. For that reason, further studies on the *Pulmonaria*–derived extracts, covering analyses of the digested extracts and in vivo assays are needed.

## 4. Materials and Methods

### 4.1. Reagents

Chloroform, methanol (isocratic grade) and acetonitrile (LC–MS grade) were purchased from Merck (Darmstadt, Germany). Formic acid (LC–MS grade) and digoxin (internal standard, IS) were purchased from Sigma–Aldrich (St. Louis, MO, USA). Ultrapure water was prepared using a Milli–Q water purification system (Millipore, Milford, CT, USA). Peroxynitrite was synthesized according to the method of Pryor et al. [40]. DPPH, Trolox^®^, rosmarinic acid, thiobarbituric acid, Histopaque^®^–1077 medium, indomethacin, penicillin–streptomycin solution for cell culture and resazurin (In vitro toxicology assay kit, Resazurin based, TOX8–1KT) were also purchased from Sigma–Aldrich (St. Louis, MO, USA). COX (human) Inhibitor Screening Assay Kit (Item No. 701230) and COX Colorimetric Inhibitor Screening Assay Kit (Item No. 701050) were from Cayman Chemicals (Ann Arbor, MI, USA). Reagents and cuvettes for cytotoxicity assays were purchased from NanoEnTek Inc. (Seoul, Korea). All other reagents were provided by local or international suppliers (mainly Avantor Performance Materials, Gliwice, Poland).

### 4.2. Plant Material

Aerial parts (early spring flowering shoots) of *P. obscura* Dumort. were collected from natural habitat in Puławy (N51°24′42.4” E21°57′30”), whereas aerial parts of *P. officinalis* L. were gathered from the national herb producer (Kania, Częstochowa, Poland). The voucher samples of both species (POBSC./EXTR/NH/2013/1) and (POFF./EXTR/2013/1) respectively have been deposited at the Department of Biochemistry and Crop Quality, Institute of Soil Science and Plant Cultivation, Puławy, Poland.

### 4.3. Preparation and Quantitative Analysis of P. obscura and P. officinalis Phenolic–Rich Fractions

Preparation and quantitative analysis of the examined plant extracts, i.e., *P. obscura* and *P. officinalis* phenolic–rich fractions were executed according to our previously developed procedures [16]. Defatted plant material was extracted twice with 80% aq. methanol (*v*/*v*), at room temperature for 24 h, using the ultrasonic bath. The obtained extracts were filtered, concentrated under reduced pressure and lyophilized. Crude extracts of *P. obscura* and *P. officinalis* were fractionated using solid phase extraction on RP–C18 column (80 × 100 mm, Cosmosil 140C18–PREP, 140 µm; NacalaiTesque, INC., Kyoto, Japan). Each of the extracts was firstly dissolved in 1% MeOH (*v*/*v*) and independently loaded on the preconditioned RP–C18 column. 1% MeOH was also applied to remove polar constituents, while a phenolics–rich fraction was eluted using 50% MeOH (*v*/*v*). Methanol was then removed under reduced pressure, and the residual material was freeze–dried (Gamma 2–16 LSC, Christ, Osterode am Harz, Germany) and used for biological activity tests.

Determination of specialized metabolites in obtained fractions of both *Pulmonaria* species was carried out as described previously [16], using the Thermo Scientific Ultimate 3000 RS (Thermo Fischer Scientific, Waltham, MA, USA) liquid chromatography (LC) system, coupled with a Bruker Impact II HD (Bruker, Billerica, MA, USA) electrospray ionization high–resolution quadrupole–time–of–flight mass spectrometer (ESI–HR Q–TOF–MS) and the same analytical procedure.

For analyses of biological activity, stock solutions of the examined *Pulmonaria* phenolic–rich fractions and rosmarinic acid (a reference compound) were prepared using 25% DMSO; the stock solution of Trolox was dissolved in 50% ethanol. The final concentration of these solvents in experimental systems, including the Evans Blue solution, blood plasma, platelet–rich plasma (PRP), or PBMCs suspensions amounted to 0.025% and 0.05%, respectively.

### 4.4. Peroxynitrite (ONOO^−^) Scavenging Assay

The assay was carried out according to our previously described protocol [45]. Briefly, the method was based on the prevention of peroxynitrite–mediated oxidation of Evans blue dye by the examined substances. Their ONOO^−^–scavenging abilities were determined indirectly, i.e., by measurements of the inhibition of Evans blue bleaching (at λ = 608 nm). A decrease of the Evans dye color, induced by 1 mM ONOO^−^, was calculated using the following equation: % of sample bleaching = 100 × (A0–A1)/A0. The absorbance of control samples (untreated with ONOO^−^) was assumed as A0 value, while A1 was the absorbance recorded after 30 min of incubation of reaction mixtures (containing 1 mM ONOO^−^ and the investigated *Pulmonaria* extracts (1–100 µg/mL), or reference substances (1–50 µg/mL)). Results obtained for samples treated with ONOO^−^– in the absence of the antioxidants were then assumed as 100% of Evans Blue dye oxidation (bleaching).

### 4.5. Preparation of Blood Plasma Samples

Human blood was purchased from the Regional Centre of Blood Donation and Blood Treatment in Lodz, Poland. All blood units were commercially available. The study was approved by the committee on the Ethics of Research at the University of Lodz, Poland (Protocol No. 9/KBBN–UŁ/II/2016).

In the antioxidant part of the study, rosmarinic acid and Trolox were used as positive controls. Blood plasma samples were pre–incubated for 15 min, at room temperature, with the *Pulmonaria* fractions (1–100 µg/mL) or reference antioxidants. For the evaluation of antioxidant actions of all these substances in blood plasma, the samples were exposed to ONOO^−^, which was added to the final concentrations of 100 µM (in the 3–nitrotyrosine (3–NT), thiobarbituric acid–reactive substances (TBARS) and lipid hydroperoxide assays) or 150 µM (in the non-enzymatic antioxidant capacity assay, NEAC). Samples containing plasma treated with ONOO^−^ in the absence of the *Pulmonaria* fractions were also prepared. Control plasma was treated with neither the investigated extracts/reference antioxidants nor ONOO^−^; however, it contained 0.025% DMSO (a vehicle for the extracts).

### 4.6. Determination of Antioxidant Capacity of Blood Plasma Using DPPH^•^ Assay

The assay was based on the evaluation of the non-enzymatic antioxidant capacity (NEAC) of blood plasma under the 150 µM ONOO^−^–induced oxidative stress [46,47]. For the assay, 20 µL of blood plasma was diluted using 380 µL of 0.05 M phosphate–buffered saline (PBS, pH 7.4) The stock solution of DPPH^•^ (500 µM) in methanol was diluted in this alcohol for obtaining a working reagent, with initial absorbance of 1.2 (at 517 nm). Then, 400 μL of the diluted blood plasma was mixed with 400 µL of the working solution of DPPH^•^. Reagent mixtures were incubated for 30 min, at room temperature. After centrifugation, the absorbance of clear supernatants was measured and compared with the background sample (containing 400 µL of PBS + 400 µL of DPPH^•^ working solution). The antioxidant capacity of blood plasma was calculated using the following equation: the DPPH^•^ scavenging ability = (A0–A1)/A0. The initial absorbance of DPPH^•^ solution was assumed as A0, and the A1 was absorbance obtained for the examined samples. The DPPH^•^ scavenging ability of control (untreated) plasma was assumed as 100% of NEAC.

### 4.7. Determination of Lipid Peroxidation Biomarkers in Blood Plasma Exposed to 100 µM ONOO^−^

The determination of hydroperoxide level in blood plasma was performed using the FOX–1 (ferric–xylenol orange) protocol [48], while the TBARS were assayed according to the method described by Wachowicz [49]. Rosmarinic acid and Trolox^®^ (5 µg/mL) were used as reference antioxidants.

### 4.8. Immunodetection of 3–NT in Blood Plasma and Experimental System of the Isolated Fibrinogen Preparation

3–NT was used as a biomarker of the 100 µM ONOO^−^–induced damage to blood plasma proteins. Experiments were conducted using two biological models, i.e., blood plasma and the isolated human fibrinogen (2 mg/mL, in 0.01 M Tris/HCl buffer, pH 7.4). The pre–incubation of blood plasma or fibrinogen (15 min, at room temperature) with the examined *Pulmonaria* extracts (i.e., 50% methanolic fractions; 1–100 µg/mL) or reference oxidants was followed by exposure to peroxynitrite (100 µM). The immunodetection of 3–NT was performed using a competitive enzyme–linked immunosorbent assay (ELISA), as described previously [50].

### 4.9. Evaluation of COX–2 Inhibitory Effects

Chemical reactions of the applied ELISA involved the COX–catalyzed metabolism of arachidonic acid, yielding PGF_2_α, a product of the reduction of prostaglandin H_2_ (PGH_2_). In addition to ELISA, abilities of the *Pulmonaria*–derived phenolic–rich fractions to inhibit COX–2 were confirmed in a colorimetric test, measuring the enzymatic activity of peroxidase component of COX, responsible for the oxidation of the *N*,*N*,*N*′,*N*′–tetramethyl–*p*–phenylenediamine (TMPD) chromogenic substrate. The positive control was indomethacin, a well–known non-steroidal anti-inflammatory drug. Inhibitory actions of the plant extracts and a reference COX inhibitor (indomethacin) were examined at their final concentrations of 1–100 µg/mL, following the manufacturer’s protocols. Assays were carried out in triplicate or quadruplicate.

Both the COX–2 activity and the IC_50_ values were calculated following directions and calculation patterns provided by a manufacturer of the used reagent kits. The activity of control (untreated) sample was assumed as 100% (of the maximum of antibody binding or the enzyme activity in the ELISA test and colorimetric assay, respectively). The ELISA: the maximum–binding well (B_0_) absorbance was averaged. Then, the %B/B_0_ (% of sample or standard bound/maximum bound) ratio was calculated (i.e., ratio of the assayed sample or standard absorbance to the B_0_ (maximum bound) well absorbance). The IC_50_ was calculated from a standard curve, constructed as the plot %B/B_0_ versus the concentration of the analyzed extracts or reference compound (indomethacin). In the colorimetric assay, the absorbance was averaged within each type of samples; the absorbance of the examined sample was subtracted from the absorbance of the 100% activity sample (control/untreated sample) and multiplied by 100 in order to obtain the percent inhibition. The IC_50_ was estimated using a standard curve constructed from the percent inhibition data versus the concentration of the analyzed extracts or reference compound.

### 4.10. Cytotoxicity Assays

Cytotoxicity of the examined *Pulmonaria* fractions was evaluated using two experimental models: peripheral blood mononuclear cells and blood platelets. PBMCs were isolated from fresh human blood, according to the protocol provided by the manufacturer. In preliminary tests, a direct influence of the examined extracts on PBMCs was assessed in cells suspended only in phosphate–buffered saline (0.02 M PBS, containing 1 × 10^6^ of cells/mL) and incubated with the examined plant extracts (1–100 µg/mL) for 4 h, at 37 °C. This direct exposure of PBMCs to the *Pulmonaria* preparations quickly provided data if these extracts might generate damage to cell membranes. Then, cell viability (%) was estimated spectrofluorometrically, in a microchip–type automatic cell counter Adam–MC DigitalBio (NanoEnTek Inc., Seoul, Korea), using propidium iodide as a fluorescent dye.

Effects of the *Pulmonaria* phenolic–rich fractions on PBMCs viability were also evaluated employing the resazurin metabolism assay after 24 h of incubation. In this test, PBMCs were suspended in RPMI–1640 medium (1.5 × 10^6^ of cells/mL) and incubated in 96–well microplates with the examined *Pulmonaria* fractions (1–100 µg/mL) for 24 h, in 37 °C, at 5% of CO_2_ concentration and 95% humidity. After the incubation, the resazurin solution was added (to the final concentration of 10%). Cell viability was recorded after 4 h of incubation with resazurin under analogous conditions, using a microplate spectrophotometer BMG Labtech SectroStarNano, at λ = 600 nm (690 nm was a reference wavelength).

Additionally, the possibility of toxic action of the examined extracts towards blood platelets was evaluated, based on measurements of lactate dehydrogenase (LDH) activity in platelet–poor plasma (PPP, a physiological medium of blood platelets). Since blood platelets are very susceptible to the action of different exogenous substances, these cells are used as a trustworthy indicator of cytotoxicity of different compounds, including plant extracts. The assay was carried out according to a previously described protocol [51]. Briefly, platelet–rich plasma (PRP) was preincubated for 30 min (at 37 °C) with the investigated fractions (1–100 µg/mL). Then, PRP samples were centrifuged, and clear supernatants of PPP were collected. The LDH leakage was determined and expressed as a percentage of LDH activity recorded in control samples (obtained from PRP, untreated with the examined *Pulmonaria* phenolic–rich fractions).

### 4.11. In Silico Study: Prediction of Bioactivity and Docking

The prediction of bioactivity and drug–likeness properties of main components of the examined *Pulmonaria* phenolic–rich fractions were performed using Molinspiration Cheminformatics website–calculation of Molecular Properties and Bioactivity Score–Predict Bioactivity tool (http://www.molinspiration.com/cgi–bin/properties). Dockings of the most common compounds detected in *Pulmonaria* fractions to COX–2 crystal structure were performed in Autodock Vina 1.1.2 (http://vina.scripps.edu/) [52]. Coordinates of COX–2 4 COX [53], which contains bound indomethacin, were downloaded from RCSB Protein Data Bank (http://www.rcsb.org/) [53]. The PDB file was deprived manually of all HETATM atoms including inhibitor atoms to free its binding site. Additionally, indomethacin structure was implemented as a reference compound and was also docked to calculate its binding enthalpy and to compare with the crystal structure. 3D ligand structures were found on PubChem (https://pubchem.ncbi.nlm.nih.gov/) or ChemSpider (http://www.chemspider.com/) websites and converted to MOL2 format using Open Babel (http://openbabel.org). Geometries of the ligand structures were optimized in Avogadro (http://avogadro.cc) [54] using the MMFF94 force field [55]. The coordinates of the ligands and the protein structures were prepared properly for docking in ADT software (http://autodock.scripps.edu/resources/adt) [56]. 10–fold dockings and thereafter parsing of affinity energy for all compounds were automated by scripts written in Python. Autodock Vina docking volume of 4COX covered boxes of two similar, opposite indomethacin binding places with center coordinates x, y, z: 24.864, 24.048, 10.330 and 69.785, 20.297, 7.825, respectively. The dimensions of both cubes were established as 26, 26, 26 covering the active sites of the enzyme molecules. Visualization of the docking poses with protein was prepared in UCSF Chimera 1.10 (http://www.cgl.ucsf.edu/chimera/) [57].

### 4.12. Statistical Analysis

The first step of statistical analysis included the elimination of the uncertain data by the Q–Dixon test. The statistical significance was evaluated using the Dunnett’s test or Student’s (with Bonferroni correction) *t*-test. All assays were done at least in duplicate (at least two independent pre–incubations of the *Pulmonaria* phenolic–rich fractions with plasma, isolated fibrinogen preparations, PBMCs, or PRP from each donor were performed). Values in this study are expressed as mean ± SD; *p* < 0.05 was assumed as statistically significant; *n* = number of independent experiments/blood donors.

## 5. Conclusions

The present study provides new information on antioxidant and the COX–2–inhibitory activities of plant phenolic–rich fractions, derived from two *Pulmonaria* species. Both of the examined fractions displayed comparable antioxidant properties, but the COX–2 inhibitor screening clearly indicated on higher inhibitory potential of *P. officinalis*. In addition to literature data on the anti-inflammatory activity of rosmarinic acid, the in silico part of this study demonstrated that also other components of the examined plant extracts displayed the COX–2 –inhibitory potential. General insight into results of in silico studies and analyses of the phytochemical profile of both extracts suggest that the *Pulmonaria* phytochemicals, especially salvianolic acid H and menisdaurin may contribute to the COX–2 inhibitory effects of *P. officinalis*. These observations may be an interesting background for further studies on the anti-inflammatory potential of *Pulmonaria* plants.

## Figures and Tables

**Table 1:** Phytochemical composition of the examined phenolic-rich fractions isolated from *P. obscura* and *P. officinalis*.

Content of Phytochemicals (% of the Fraction)		
	*P. obscura*	*P. officinalis*
Phenolic acids	81.01	81.15
Lignans	11.27	6.46
Flavonol glycosides	4.95	5.16
Others	2.36	7.22

**Table 2:** Metabolite content in the examined phenolic-rich fractions, isolated from aerial parts of *P. obscura* and *P. officinalis*.

No.	Compound Name	Contents [µg/mg of Fraction] (Mean ± SD, *n* = 3)	
		*P. obscura*	*P. officinalis*
1	Danshensu	2.40 ± 0.10	1.52 ± 0.06
2	Menisdaurin	16.36 ± 0.43	39.08 ± 1.56
3	3-*O*-(*E*)-caffeoyl-threonic acid	1.18 ± 0.10	1.53 ± 0.05
4	2-*O*-(*E*)-caffeoyl-l-threonic acid	3.34 ± 0.11	4.52 ± 0.14
5	Lycoperodine-1	Traces	1.20 ± 0.04
6	Chlorogenic acid	4.87 ± 0.13	4.32 ± 0.11
7	Actinidioionoside	Traces	Traces
8	Caffeic acid	Traces	2.31 ± 0.11
9	Cryptochlorogenic acid	0.74 ± 0.03	0.22 ± 0.01
10	3’-*O*-(*E*)-feruloyl-α-sorbopyranosyl-(2’→1)-α-glucopyranoside	Traces	0.45 ± 0.01
11	2-*O*-(*E*)-caffeoyl-d-glyceric acid	1.24 ± 0.02	5.10 ± 0.14
12	4-*O*-(*E*)-caffeoyl-l-threonic acid	1.50 ± 0.08	2.78 ± 0.07
13	Neochlorogenic acid	0.37 ± 0.01	0.04 ± 0.00
14	3-*O*-(*E*)-caffeoyl-glyceric acid	Traces	2.08 ± 0.06
15	3-*O*-*p*-coumaroylquinic acid	0.87 ± 0.03	2.29 ± 0.04
16	4-*O*-*p*-coumaroylquinic acid	Traces	Traces
17	5*-O*-*p*-coumaroylquinic acid	0.93 ± 0.04	1.88 ± 0.04
18	Globoidnan B	29.79 ± 4.46	24.16 ± 3.33
19	Rutin	Traces	Traces
20	Nicotiflorin isomer	Traces	Traces
21	Quercetin 3-*O*-*β*-glucoside	0.62 ± 0.01	0.33 ± 0.02
22	Yunnaneic acid E	27.32 ± 0.23	12.07 ± 0.33
23	Quercetin 3-*O*-(6′′-*O*-malonyl)-*β*-glucoside	4.10 ± 0.18	2.82 ± 0.15
24	Nicotiflorin	Traces	Traces
25	Astragalin	Traces	Traces
26	Shimobashiric acid C	7.46 ± 0.17	5.46 ± 0.17
27	Rosmarinic acid	301.46 ± 35.04	159.28 ± 17.09
28	Kaempferol 3*-O*-(6′′-*O*-malonyl)-*β*-glucoside	0.40 ± 0.08	Traces
29	Monardic acid A	42.99 ± 5.04	17.56 ± 2.77
30	Yunnaneic acid E-1	Traces	Traces
31	Lithospermic acid A	80.13 ±6.44	44.20 ± 0.05
32	Pulmonarioside A	Traces	Traces
33	Salvianolic acid H	8.97 ± 0.82	26.23 ± 0.75
34	Lithospermic acid B	NA	NA
35	Pulmonarioside B	0.50 ± 0.03	Traces
36	Yunnaneic acid B	23.65 ± 0.52	22.65 ± 1.49
37	Globoidnan A	4.10 ± 0.17	0.57 ± 0.02
38	Pulmitric acid A	Traces	Traces
39	Pulmitric acid B	Traces	Traces
40	Isosalvianolic acid A	Traces	Traces
41	Isosalvianolic acid A-1	Traces	Traces
42	Isosalvianolic acid A isomer	Traces	Traces
43	Rosmarinic acid methyl ester	Traces	Traces
44	Salvianolic acid H-9′′-methylester	3.87 ± 0.11	0.79 ± 0.02
45	Lycopic acid C	NA	NA

TR–traces, indicates level below the limit of quantification, NA–not analyzed, ND–not detected.

**Table 3:** Determination of the ONOO^−^ scavenging abilities of *P. obscura* and *P. officinalis* phenolic-rich fractions, based on their inhibitory effects on the 1 mM ONOO^−^-induced bleaching (peroxidation) of Evans blue dye (*n* = 4–6).

Inhibition of the ONOO^−^-Induced Evans Blue Peroxidation	
	IC_50_ [µg/mL]
*P. obscura*	36.71
*P. officinalis*	32.66
Rosmarinic acid	13.34
Trolox	2.76

**Table 4:** Protective effects of the examined *Pulmonaria* phenolic-rich fractions on the 100 µM ONOO^−^-induced nitration of tyrosine residues in human blood plasma proteins and the isolated human fibrinogen (* *p* < 0.05; ** *p* < 0.01; *** *p* < 0.001 for plasma treated with ONOO^−^ in the absence of examined substances vs. plasma treated with ONOO^−^ in the presence of the extracts; ^###^ *p* < 0.001 for the control samples vs. samples treated with ONOO^−^ in the absence of examined substances; /-per; *n* = 7).

*Pulmonaria* Phenolic-Rich Fractions and Reference Compounds [µg/mL]			3-Nitrotyrosine [nmol 3-NT-Fg/mg of Protein]	
			Isolated Fibrinogen	Blood Plasma
control (untreated) samples		0	0.006 ± 0.003	0.026 ± 0.008
samples treated with ONOO^−^ in the absence of examined substances		0	1.326 ± 0.072 ^###^	2.605 ± 0.392 ^###^
samples treated with ONOO^−^ in the presence of:	*P. obscura*	1	1.357 ± 0.058	2.152 ± 0.640
		5	1.200 ± 0.158	2.040 ± 0.520
		25	0.889 ± 0.259 **	1.881 ± 0.709 **
		50	0.785 ± 0.354 *	1.590 ± 0.562 ***
		100	0.620 ± 0.459 ***	1.060 ± 0.539 ***
	*P. officinalis*	1	1.257 ± 0.111	2.070 ± 0.207 **
		5	1.106 ± 0.279	2.335 ± 0.379
		25	0.895 ± 0.244 *	2.301 ± 0.559
		50	0.917 ± 0.280 *	1.342 ± 0.401 **
		100	0.799 ± 0.387 **	0.943 ± 0.317 ***
	Rosmarinic acid	5	0.803 ± 0.411 **	2.069 ± 0.308 **
	Trolox	5	0.898 ± 0.328 *	1.788 ± 0.793 **

**Table 5:** Protective effect of *Pulmonaria* phenolic-rich fractions on the 100 µM ONOO^−^-induced peroxidation of blood plasma lipids. The peroxidation of blood plasma lipids in samples treated with ONOO^−^ in the absence of the examined substances was assumed as 100% of lipid peroxidation (* *p* < 0.05; ** *p* < 0.01; *** *p* < 0.001; *n* = 8).

*Pulmonaria* Phenolic-Rich Fractions and Reference Compounds [µg/mL]			Plasma Lipid Peroxidation Biomarkers	
			% of Lipid Hydroperoxides Generation	% of TBARS Formation
plasma treated with ONOO^−^ in the presence of:	*P. obscura*	1	72.924 ± 6.660 **	96.234 ± 6.746
		5	65.102 ± 6.242 **	83.308 ± 8.987 *
		25	54.075 ± 5.957 ***	71.977 ± 14.638 *
		50	54.983 ± 6.966 ***	62.389 ± 10.826 ***
		100	53.805 ± 9.557 ***	63.759 ± 9.306 ***
	*P. officinalis*	1	75.492 ± 6.788 **	84.685 ± 13.169
		5	70.085 ± 6.394 **	79.188 ± 11.122 *
		25	59.695 ± 5.208 **	75.782 ± 10.019 *
		50	59.927 ± 8.141 ***	66.851 ± 12.462 **
		100	57.126 ± 8.145 ***	57.746 ± 8.855 ***
	Rosmarinic acid	5	58.246 ± 14.864 **	67.552 ± 11.641 **
	Trolox	5	63.986 ± 7.585 ***	69.212 ± 11.459 ***

**Table 6:** The IC_50_ values established during a screening of inhibitory actions of *P. obscura* and *P. officinalis* phenolic-rich fractions on COX-2 activity (*n* = 3–4).

The Examined Substances	IC_50_ [µg/mL]	
	The PGF_2α_Generation (ELISA)	The Oxidation of Chromogenic Substrate (Colorimetric Assay)
*P. obscura*	51.00	58.59
*P. officinalis*	7.24	13.28
Indomethacin	0.05	2.06

**Table 7:** Assessment of cytotoxicity of the examined *Pulmonaria* phenolic-rich fractions towards peripheral blood mononuclear cells and blood platelets in vitro. Viability of control (untreated) PBMCs and LDH leakage in control blood platelets were assumed as 100%; (*n* = 7–13; *p* > 0.05).

	*Pulmonaria* Phenolic-Rich Fraction Concentration [µg/mL]	% Viability of PBMCs Based on Propidium Iodide Assay	% Viability of PBMCs Based on Resazurin Reduction Assay	% of LDH Leakage from Blood Platelets
*P. obscura*	1	102.15 ± 13.12	96.84 ± 8.70	100.55 ± 9.04
	5	106.80 ± 6.21	94.59 ± 8.14	99.67 ± 12.50
	25	100.11 ± 9.78	95.25 ± 8.68	101.16 ± 11.22
	50	105.45 ± 6.52	98.53 ± 11.10	97.86 ± 6.98
	100	100.21 ± 12.08	93.75 ± 8.69	94.92 ± 6.84
*P. officinalis*	1	102.97 ± 9.32	93.71 ± 8.10	103.30 ± 13.33
	5	106.66 ± 7.05	93.76 ± 10.54	105.59 ± 15.16
	25	104.18 ± 10.77	93.59 ± 9.32	102.32 ± 7.57
	50	104.67 ± 9.15	96.70 ± 7.51	98.68 ± 13.30
	100	107.05 ± 5.53	97.33 ± 9.43	99.65 ± 7.67

**Table 8:** Summary of computational analysis of the most abundant compounds found in *P. obscura* and *P*. *officinalis*. MW–predicted molecular weight, MBS, HA–the number of heavy atoms, PI–Protease inhibitor Molinspiration bioactivity score v2014.03, MBS EI–Enzyme inhibitor Molinspiration bioactivity score v2014.03, ΔG°–predicted standard free energy of ligand binding, LE-Ligand Efficiency (LE = –RTlnK_d_/HA or –ΔG°/HA), LELP = milog P/LE. The most probable inhibitors and indomethacin are marked in bold. Locations of docking of two the most potent COX inhibitors, i.e., menisdaurin and salvianolic acid H as well as indomethacin (a reference COX inhibitor) are visualized in Figure 3.

(No.)	Compound Names, Chemical Formula and SMILES	~MW (Da)	H.A.	milogP	MBS PI	MBS EI	ΔG°*_bind_* (kcal/mol) COX2	LE LELP
(**4**)	**2–O–*E*–caffeoyl–l–threonic acid** O=C(C=Cc1ccc(O)c(O)c1)O[C@H](C(=O)O)[C@@H](O)CO	298	21	−0.49	−0.13	**0.34**	−7.6 ± 0.1	0.36 –1.36
(**1**)	Danshensu c1(ccc(c(c1)O)O)C[C@@H](O)C(=O)O	198	14	−0.25	−0.27	0.13	−6.8 ± 0.1	0.49 –0.51
(**6**)	**Chlorogenic acid** O=C(C=Cc1ccc(O)c(O)c1)O[C@@H]2C[C@](O)(C(=O)O)C[C@@H](O)[C@H]2O	354	25	−0.45	**0.25**	**0.62**	−7.9 ± 0.2	0.32 –1.41
(**37**)	**Globoidnan A** c1(cc2cc(c(cc2c(c1)c1ccc(c(c1)O)O)O)O)C(=O)O[C@@H](C(=O)O)Cc1ccc(c(c1)O)O	492	36	3.12	0.11	**0.26**	−8.7 ± 0.3	0.24 13.00
(**18**)	**Globoidnan B** O([C@H](Cc1cc(c(cc1)O)O)C(=O)O)C(=O)c1c(c(c2c(c1)cc(c(c2)O)O)c1cc(c(cc1)O)O)C(=O)O	537	39	2.28	0.09	**0.28**	−6.9 ± 0.2	0.18 12.67
(**31**)	**Lithospermic acid A** c12[C@@H]([C@@H](Oc1c(ccc2/C=C\C(=O)O[C@@H](Cc1cc(c(cc1)O)O)C(=O)O)O)c1cc(c(cc1)O)O)C(=O)O	539	39	1.57	0.06	**0.28**	−7.8± 0.1	0.20 7.85
(**2**)	**Menisdaurin** O1[C@@H]([C@H]([C@@H]([C@H]([C@@H]1O[C@H]1/C(=C\C#N)/C=C[C@H](C1)O)O)O)O)CO	313	22	−1.79	**0.28**	**0.91**	−6.2 ± 0.2	0.28 –6.39
(**29**)	**Monardic acid A** O1[C@@H]([C@H](c2c1c(ccc2/C=C/C(=O)O[C@@H](Cc1cc(c(cc1)O)O)C(=O)O)O)C(=O)O)c1cc(c(cc1)O)O	539	39	1.57	0.06	**0.28**	−7.6 ± 0.5	0.20 7.85
(**27**)	**Rosmarinic acid** [C@@H](C(=O)O)(OC(=O)/C=C/c1ccc(c(c1)O)O)Cc1ccc(c(c1)O)O	360	26	1.53	0.15	**0.24**	−8.3 ± 0.1	0.32 4.78
(**23**)	**Quercetin 3–*O*–(6’’–*O*–malonyl)–β–glucoside** O1[C@@H]([C@H]([C@@H]([C@H]([C@@H]1Oc1c(oc2c(c1=O)c(cc(c2)O)O)c1cc(c(cc1)O)O)O)O)O)COC(=O)CC(=O)O	550	39	−0.66	−0.03	**0.35**	−6.3 ± 0.3	0.16 –4.13
(**26**)	Shimobashiric acid C [C@@H]1([C@@H]([C@@H]([C@H]1c1cc(c(cc1)O)O)C(=O)O[C@@H](C(=O)O)Cc1cc(c(cc1)O)O)c1cc(c(cc1)O)O)C(=O)O[C@@H](C(=O)O)Cc1cc(c(cc1)O)O	721	52	2.98	−0.52	−1.21	−3.6 ± 0.7	0.07 42.57
(**33**)	**Salvianolic acid H** O([C@H](Cc1cc(c(cc1)O)O)C(=O)O)C(=O)/C=C/c1c(c(c(cc1)O)O)/C=C/c1cc(c(cc1)O)O	495	36	3.01	0.08	**0.21**	−8.8 ± 0.4	0.24 12.54
(**22**)	Yunnaneic acid E [C@@H]1([C@H](C(=C[C@H]([C@@H]1c1ccc(c(c1)O)O)C(=O)O)/C=C/C(=O)O[C@@H](C(=O)O)Cc1ccc(c(c1)O)O)C(=O)O)C(=O)O	573	41	1.03	−0.01	0.06	−7.7 ± 0.3	0.19 5.42
(**36**)	Yunnaneic acid B O1[C@]2([C@@](O[C@@]31[C@H]1[C@@H]([C@H]([C@@H](C3=O)[C@H](C1)CCC(=O)O[C@@H](C(=O)O)Cc1cc(c(cc1)O)O)C(=O)O)c1cc(c(cc1)O)O)([C@H]1[C@@H]([C@H]([C@@H]2[C@H](C1)/C=C/C(=O)O[C@@H](C(=O)O)Cc1cc(c(cc1)O)O)C(=O)O)c1cc(c(cc1)O)O)O)O	1001	79	1.95	−3.72	−3.78	−10.2 ± 0.1	0.13 15
	**Indomethacin** C(=O)(n1c(C)c(c2cc(ccc12)OC)CC(=O)O)c1ccc(Cl)cc1	358	25	3.99	−0.11	**0.30**	−9.4 ± 1.3	0.38 10.50

## Data Availability

Not applicable.

## Sample Availability

Samples of the extracts are available from the authors.
